# Monitoring the Return to Sport Transition After ACL Injury: An Alpine Ski Racing Case Study

**DOI:** 10.3389/fspor.2020.00012

**Published:** 2020-03-03

**Authors:** Matthew J. Jordan, Nathaniel Morris, Mike Lane, Jeremiah Barnert, Katie MacGregor, Mark Heard, Sarah Robinson, Walter Herzog

**Affiliations:** ^1^Canadian Sport Institute Calgary, Calgary, AB, Canada; ^2^Faculty of Kinesiology, The University of Calgary, Calgary, AB, Canada; ^3^Banff Sports Medicine Centre, Banff, AB, Canada

**Keywords:** knee injuries, training load, vertical jump asymmetry, vertical jump power, quadriceps/hamstrings strength, ACL reinjury

## Abstract

Alpine ski racing is an extreme sport and ski racers are at high risk for ACL injury. ACL injury impairs neuromuscular function and psychological readiness putting alpine skiers with ACL injury at high risk for ACL reinjury. Consequently, return to sport training and testing protocols are recommended to safeguard ACL injured athletes against reinjury. The aim of this paper was to present a real-world example of a return to sport training plan for a female elite alpine ski racer who sustained an ACL injury that was supported by an interdisciplinary performance team (IPT) alongside neuromuscular testing and athlete monitoring. A multi-faceted return to sport training plan was developed by the IPT shortly after the injury event that accounted for the logistics, healing, psychological readiness, functional milestones, work capacity and progression to support the return to sport/return to performance transition. Neuromuscular testing was conducted at several timepoints post-injury. Importantly, numerous pre-injury tests provided a baseline for comparison throughout the recovery process. Movement competencies and neuromuscular function were assessed, including an evaluation of muscle properties (e.g., the force-velocity and force-length relationships) to assist the IPT in pinpointing trainable deficits and managing the complexities of the return to sport transition. While the athlete returned to snow 7 months post-injury, presenting with interlimb asymmetries below 10%, functional and strength deficits persisted up to 18 months post-injury. More research is required to establish a valid return to sport protocol for alpine ski racers with ACL injury to safeguard against the high risk for ACL reinjury.

## Introduction

An anterior cruciate ligament (ACL) tear is the most common injury amongst alpine ski racers (Flørenes et al., 2009; Bere et al., 2014), and the injury rate has remained high despite injury prevention efforts (Haaland et al., 2016). ACL reconstruction (ACLR) surgery is often recommended to alpine skiers who have sustained an ACL injury to restore knee joint stability (Jordan et al., 2017a). However, ≈20–30% of alpine ski racers with ACLR will subsequently suffer an ACL reinjury, often to the contralateral limb and within 2 years post-surgery (Pujol et al., 2007; Haida et al., 2016; Jordan et al., 2017c). ACL tears in alpine ski racing are typically severe and occur alongside combined injury to other knee joint structures (e.g., multi-ligament injury, articular cartilage injury, meniscal tears), which may accelerate the development of early onset knee joint osteoarthritis (Jordan et al., 2017c).

It is estimated that between 50 and 60% of athletes who sustain an ACL injury will return to competitive sport (Ardern et al., 2014; Webster et al., 2019), while the proportion of alpine skiers returning to competitive sport may be even higher (Haida et al., 2016). Athletes who undergo ACLR surgery require extensive rehabilitation and training to restore knee joint function and objectively determined functional criteria are recommended to guide the return to sport transition to safeguard against secondary ACL injury (Buckthorpe, 2019). However, sports medicine practitioners and trainers often rely solely on time-based criteria to determine when an athlete is sufficiently prepared to return to sport (Barber-Westin and Noyes, 2011), and it has been shown that only a small fraction of ACLR athletes (23%) pass objectively determined functional criteria prior to return to sport (Webster and Hewett, 2019).

Further, passing commonly conducted return to sport testing batteries that include criteria like achieving >90% limb symmetry in single leg hop tests, appear to provide little assurance that an athlete is physically prepared for return to sport after ACLR to avoid a second ACL injury (Webster and Hewett, 2019). A prospective study evaluating a battery of return to sport tests after ACLR found no association between achieving a limb symmetry index >90% in various functional tests, such as the single leg hop for distance, single leg triple hop, and 6 m timed hop, and a reduction in ACL reinjury rates (Grindem et al., 2016). Only quadriceps strength symmetry, and delaying return to sport in the first 9 months post-surgery, were associated with a reduction in the risk for ACL reinjury. These findings suggest that ACLR athletes may compensate in return to sport functional tests to achieve the established benchmark while potentially masking deficits that put them at risk for ACL reinjury.

In addition to the prevalence of ACL reinjury (Pujol et al., 2007; Haida et al., 2016; Jordan et al., 2017a), actively competing alpine skiers with ACLR have also been shown to demonstrate functional deficits compared to non-injured alpine ski racers, including elevated interlimb asymmetry in lower limb muscle power, thigh muscle rapid force development ability (rate of force development—RFD), and thigh muscle maximal strength that persist despite returning to competitive sport (Jordan et al., 2015b,c, 2017b, 2018). The high frequency of ACL injury in alpine ski racing, the likelihood for combined injury, and the fact that many alpine skiers will attempt to return to sport after ACLR surgery, strongly suggest the importance of a sport-specific return to sport protocol to safeguard alpine skiers against ACL reinjury (Jordan et al., 2015a). Further, building resilience against ACL reinjury is multifactorial and involves not only restoring functional abilities and tissue healing but also psycho-emotional readiness (Ardern et al., 2011). Therefore, athletes returning from ACL injury may be best supported by an interdisciplinary performance team (IPT) in which domain-specific practitioners work collaboratively to support the transition back to sport (Forsdyke et al., 2017; Wang et al., 2019).

The aim of this case study is to present the details of a return to sport training and testing protocol used with a female elite alpine ski racer who sustained an ACL injury and subsequently underwent ACLR surgery. The return to sport transition presented in this case study will include discussion on the importance of objective functional assessments, workload monitoring, the restoration of work capacity and the role of the IPT in supporting alpine skiers with ACLR throughout the return to sport and return to performance transition.

## Return to Sport Testing Methodology

### Participant Characteristics

A female elite alpine ski racer (Age = 28 years) sustained a right knee injury (high grade ACL tear, medial meniscal tear, lateral tibial plateau compression fracture). She underwent an ACLR surgery using an 8.5 mm quadruple semitendinosus (ST) autograft. The Conjoint Research Ethics Board at the University of Calgary approved the experimental protocols and the participant gave written informed consent prior to her involvement in the testing protocols.

### Interdisciplinary Performance Team Personnel and Testing Environment

The athlete's IPT consisted of seven sport science/sport medicine practitioners including the following disciplines: sport science/physiology, strength & conditioning (S&C), physiotherapy, sports medicine, orthopedic surgery, mental performance, and sport nutrition/dietetics. All neuromuscular testing was undertaken in the Strength and Power laboratory at the Canadian Sport Institute Calgary by qualified exercise testing personnel. A return to sport training plan detailed the athlete's training/rehabilitation priorities and logistics supporting the rehabilitation/re-training process.

### Internal Workload Monitoring

Internal workload was assessed using the sessional rating of perceived exertion (sRPE) method (Foster, 1998). The participant was asked to record the training session type, the training session duration in minutes and the perceived exercise intensity using a modified Borg scale (1 = Easy, 10 = Maximal) for every unique training session each day. Data was collected using an online form. The session duration in minutes and perceived session intensity were multiplied to obtain the internal sessional workload in arbitrary units (AU). The weekly internal training load was then obtained by summing the sRPE workload for the total number of training sessions per week (total number of training weeks recorded from May 2012 to May 2019: *n* = 315). Logging compliance was not monitored throughout the pre-injury and post-injury time period and while the athlete demonstrated a high degree of consistency during the data collection time period, the reported workloads approximated the total workload incurred by the athlete.

### Neuromuscular Testing

#### Dual Force Plate Vertical Jump Force-Time Assessments

Countermovement (CMJ) and squat jump (SJ) force-time assessments on a dual force plate system were conducted regularly throughout the off-snow training periods in the pre-injury and post-injury periods. The standard protocols included: (1) 5-jump CMJ test using a self-determined depth; (2) 5-jump SJ test using a standardized depth of 90° of knee flexion; (3) 80 s repeated squat jump test using a standardized depth of 90° of knee flexion (total jumps per test: *n* = 20); (4) loaded CMJ test using three loading conditions including no external load, and an external load equal to ≈30% and ≈60% of body mass (Total CMJ tests conducted: *n* = 37; Total SJ tests conducted: *n* = 36; Total 80 s repeated SJ tests conducted: *n* = 6; Total loaded jump tests conducted: *n* = 6). All jump tests were performed with the hands placed firmly on the hips except for the loaded CMJ test in which the athlete held a trap bar (hex bar) whereby the external load was positioned at the level of the hip joint.

A detailed explanation of the vertical jump testing protocol and force-time analysis procedures have been described elsewhere (Jordan et al., 2015b, 2017b, 2018). Briefly, the vertical ground reaction force (Fz) from the right and left legs were measured simultaneously using a dual force plate system (Accupower Force Platform, AMTI, Watertown, Massachusetts, USA) at a sampling frequency of 1,500 Hz and recorded on a personal computer (MyoResearch Version 3.8, Noraxon, Scottsdale, Arizona, USA). Data were exported and analyzed using a custom-built computer program (Matlab R 2018b, Mathworks, Natwick, Massachusetts, USA). The velocity of the body center of mass (BCM) was obtained by time integration of the instantaneous acceleration signal (Fz/body mass – g) calculated from the total Fz, summed from the right and left limbs. Mechanical muscle power exerted on BCM was derived continuously throughout the jumping movement by calculating the instantaneous product of Fz and BCM velocity. Jump height was determined from the BCM vertical velocity at the instant of ground toe-off [Jump Height = Takeoff Velocity^2^/2g] (Linthorne, 2001).

#### Vertical Jump Force-Time Asymmetry Assessment

An interlimb vertical jump force-time asymmetry index (AI) was calculated for discrete jump phases in the SJ and CMJ. For the CMJ, the total impulse was calculated by time integration of Fz over the eccentric deceleration phase and concentric phase, respectively (Jordan et al., 2015b). For the SJ, the early takeoff phase (initiation of the SJ to the peak of Fz in the concentric phase) and the late takeoff phase (peak Fz to the point of toe-off) were determined. The right and left total impulse were compared using a 5-jump mean AI using the following formula:

$$\begin{array}{l} AI=\left(\begin{array}{c} \frac{Right\;Impulse-Left\;Impulse}{Maximum\;of\;Left\;and\;Right\;Impulse} \end{array}\right)*100 \end{array}$$

#### 80 s Repeated Squat Jump Test

A detailed description of the 80 s repeated SJ test can be found elsewhere (Jordan et al., 2017b). Briefly, the participant descended to the squat jump start position with the hands placed firmly on the hips and held this position for 4 s. A metronome timer indicated the start of the test that was set to repeat every 4 s. Following each maximal jump, the participant landed back in the squat jump start position and maintained this position until they were cued for the next jump by a strong verbal command from the tester. The participant performed 20 maximal jumps over the 80 s jump-test protocol. The SJ mean power, takeoff velocity and AI were averaged over sets of five jumps (Set 1 = Jumps 1–5; Set 2 = Jumps 6–10; Set 3 = Jumps 11–15; Set 4 = Jumps 16–20) allowing performance fatigability to be assessed by comparing the outcome measures in Set 4 with the outcome measures obtained in Set 1.

#### Loaded Countermovement Jump Test

A three-point takeoff velocity vs. external load profile was determined by having the participant perform 5 CMJs with and without an external load corresponding to ≈30 and 60% of body mass (García-Ramos et al., 2018). The takeoff velocity was obtained from the velocity-time curve of the BCM at the instant of ground toe-off. The net eccentric deceleration impulse was also calculated by time integration of Fz between the point of the minimum downward velocity to the zero-velocity time point corresponding to the lowest position of the BCM prior to the initiation of the ascent phase. Subsequently, the line of best fit was determined between the three data points to obtain the slope, the extrapolated maximum velocity intercept (V_0_) and the extrapolated maximum external load intercept (F_0_). The eccentric deceleration impulse vs. load profile was assessed using a polynomial line of best fit.

#### Rate of Force Development and Maximal Strength Assessments

Maximal voluntary contractions (MVCs) of isometric leg press, knee extension and knee flexion were conducted in the post-surgery time period (isometric leg press tests: *n* = 3; post-surgery time intervals: 4, 6, 17 months; isometric knee extension/flexion tests: *n* = 4; post-surgery time intervals: 6, 8, 11, and 17 months). The participant was instructed to perform all isometric testing “as fast and as hard as possible.” Isometric leg press tests were performed on a custom-built seated leg press dynamometer instrumented with a single-axis force plate (PASCO, PS-2141, California, USA**)** and force was sampled at 1,000 Hz. The participant was seated in the leg press rig and instructed to hold a steady baseline force (100 N) with feedback provided by a visual display. Based on the verbal cues of the tester, the participant then performed 3 × 3 s MVCs of isometric leg press separated by a 20 s rest period.

The isometric knee extension and flexion testing was performed on a Cybex dynamometer instrumented with a third-party load cell (Omega, LC703-500, Stamford, Connecticut, USA) and force was sampled at 1,500 Hz (MyoResearch Version 3.8, Noraxon, Scottsdale, Arizona, USA). For the knee extension trials, the participant was positioned in a seated position with the knee joint angle set at 70° of knee flexion. The tester then instructed the participant to perform 3 × 3 s MVCs of isometric knee extension separated by a 20 s rest period. The isometric knee flexion testing was performed in the same manner except the athlete was positioned in a prone position (hip flexion angle = 0°). Visual feedback and strong verbal encouragement were provided throughout the testing protocol.

The moment arm (distance from the axis of rotation to the point of force application) of the shank was obtained to calculate knee extensor and knee flexor torque. The isometric force-time (leg press) and torque-time (knee extension and flexion torque) curves were smoothed (Matlab “smooth” function using 33 ms centered moving average window). A 200 ms average around the peak value was calculated to obtain the maximum torque and maximum force. The derivative of the signal was then calculated to identify the peak RFD and rate of torque development (RTD). A 100 ms average around this timepoint was calculated to obtain the RFD and RTD.

## Observations and Outcomes of the Return to Sport Transition

### Interdisciplinary Performance Team Function

#### Building the Return to Sport Training Plan

Paralleling the periodization planning process for a macrocycle training plan used in elite sport, a return to sport training plan was developed the week following surgery by the IPT to address logistic factors (e.g., travel requirements, budget, resources, IPT meeting schedule, testing schedule), estimate the stages of tissue healing based on guidance by the orthopedic surgeon, establish the progression of functional neuromuscular milestones and provide an initial forecast for the recovery process. The ACL recovery forecast was based off an emerging dataset tracking the functional recovery of alpine skiers undergoing ACLR surgery alongside tracking of additional covariates such as the potential for associated injuries that may delay return to sport (e.g., chondral injury, meniscal tears, multi-ligament injuries) (Jordan et al., 2015a, 2017c).

**Figure 1:**
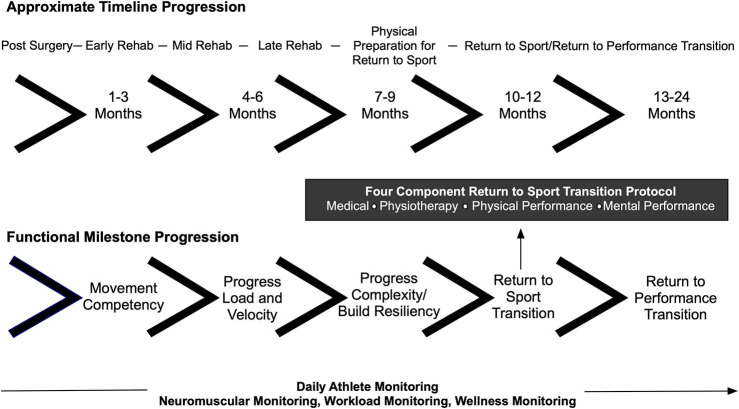
Milestone and timeline progression used in the return to sport training plan.

The return to sport training plan was segmented into the post-surgical, early rehabilitation, mid rehabilitation, late rehabilitation, physical preparation for return to sport, return to sport transition, and return to performance phases (Figure 1). Consideration was given to both the post-surgical time period reflecting the importance of tissue healing and the milestone progression for movement competencies such as lower limb energy absorption ability, a critical performance factor in alpine ski racing. The return to sport training plan also aimed to ensure the IPT-athlete-coach triad had a communication framework and accountability structure for return to sport clearance. Notably, meeting times and dates for testing were scheduled in advance prior to the commencement of the return to sport training plan.

The return to sport protocol involved four components including input from the physician, physiotherapist, physical performance staff (strength and conditioning—S&C, sport science) and mental performance coach. The return to sport protocol was communicated to all athletes and coaches at the start of each season, including the injured athlete prior to the injury event, and this was deemed an important success factor for the team approach to building and executing the return to sport training plan. To this end, the leadership structure of the IPT was characterized as a *holacracy* or a self-managed team where the domain-specific experts emerged to lead in various phases of the return to sport transition.

Twelve 4-week training mesocycles were outlined in the return to sport training plan that progressed the athlete through to the end of the return to sport transition period (≈12 months post-surgery). Each mesocycle was further subdivided into: (1) a 3-day assessment period that included a medical evaluation, neuromuscular testing, anthropometric testing, fitness testing, and a meeting with the mental performance coach; (2) an IPT debrief and program planning meeting where the findings of the assessments were integrated into a targeted training program; (3) an IPT-athlete-coach debrief meeting; (4) training program orientation sessions with the athlete that were attended by the physiotherapist, S&C coach and sport scientist enabling the training and rehabilitation plans to be adjusted if required; (5) execution of the 3-week training cycle; and finally (6) a recovery microcycle (3–5 days in duration). An athlete monitoring system that included neuromuscular monitoring, off-snow/on-snow workload monitoring and an evaluation of the athlete's recovery status (data not presented) underpinned the return to sport training plan.

#### The Interdisciplinary Performance Team's Role in Managing Uncertainty

A key function of the IPT was to manage uncertainty surrounding the return to sport transition alongside monitoring the athlete's advancement so that progressions/regressions could be made as required. Despite the best efforts of the IPT to clearly communicate the return to sport protocol, it was impossible to accurately predict the athlete's actual progression through the return to sport training plan. Return to sport after ACLR is a complex process with few certainties and implications for both erroneously accelerating or delaying an athlete's timeline for return to sport exist. To this end, objectively determined milestones and neuromuscular monitoring supported the return to sport process and permitted the IPT to manage the inherent uncertainty, high expectations, and potential barriers to a successful return to sport.

**Figure 2:**
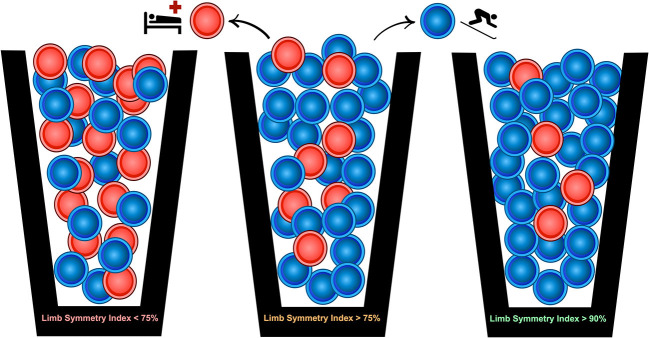
A probability-based risk profile using three different quadriceps limb symmetry index thresholds is presented to contextualize the uncertainty surrounding the return to sport transition after ACL reconstruction surgery. Thresholds were chosen to reflect known associations between the quadriceps limb symmetry index and risk of ACL reinjury (Grindem et al., 2016).

The uncertainty around return to sport decision making was communicated using a simple probability example showing an image of three buckets containing blue chips and red chips (Figure 2). The uncertainty of the return to sport transition after ACLR can be characterized by the presence of red chips and blue chips in all three buckets, differing only in terms of their relative proportions. In this example, drawing a red chip represents a reinjury while drawing a blue chip represents a successful return to sport. While red chips exist in all three buckets, the chances of drawing a red chip are smallest in the third bucket. This analogy was used to improve discussions between the athlete and IPT especially around factors such as whether or not to pursue an early return to sport.

### Workload Monitoring

**Figure 3:**
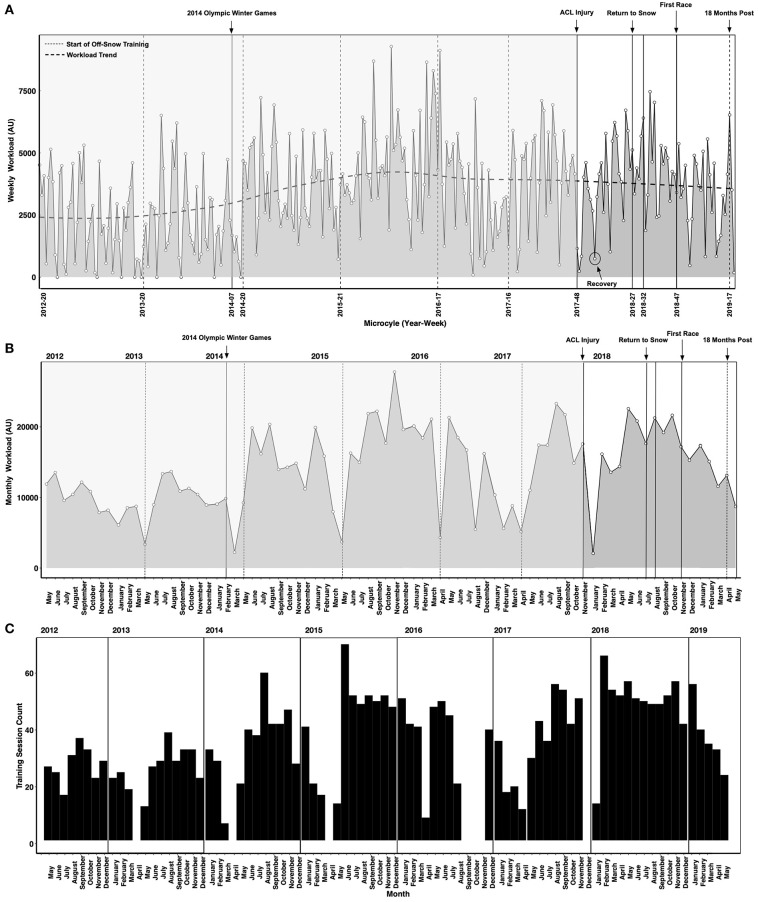
Weekly **(A)** and monthly **(B)** internal workloads (sessional rating of perceived exertion method—sRPE) in arbitrary units (AU). Vertical lines represent time points of interest. An instance of a planned recovery microcycle is highlighted (circle). **(C)** Depicts the count of logged training sessions, including periods when no logging occurred. Periods when no logging occurred are not shown in **(A,B)**.

Sessional internal workload (sRPE—AU) measurements are shown in Figure 3. While the athlete demonstrated a high degree of consistency throughout the 2012–2019 training and competition periods there were phases of the annual training cycle when workload monitoring was not performed (e.g., post-Olympic time period, post-season recovery time period, post-injury time period). In certain instances, technological limitations precluded logging (e.g., during training periods in the Southern hemisphere where internet access was limited). For example, a cessation in logging occurred between August-November of 2016 due to this factor. Nevertheless, logging throughout the post-injury period enabled the IPT to track the workload progression to ensure adequate recovery was prescribed and that the accumulated workload was sufficient to support the demands of on-snow training in the return to sport phase. The relationship between restoring sport-specific workload capacity and the risk of ACL reinjury is unclear, but it seems important to ensure that an ACLR athlete has sufficient physical reserves to support return to sport training.

Sharp decreases in workload occurred after the 2014 Olympic Winter Games due to an upper body injury sustained at the Olympics. Workloads also decreased substantially after the ACL injury sustained in late November 2017. Normal logging resumed in January 2018 and the athlete demonstrated a steady increase in workload between January 2018 and June 2018 prior to the first return to snow camp in early July 2018. Workload decreased through the return to snow/return to performance transition (October-November 2018) with the first race occurring in late November 2018. The workload composition (i.e., the workloads of different program elements such as strength training) is not shown in Figure 3A. This is a key consideration as a substantial workload was incurred due to the demands of rehabilitation. Periodic decreases in workload (i.e., recovery microcycles) can be observed throughout the rehabilitation and physical preparation phases prior to the first return to snow camp shown in Figure 3A. Rehabilitation after serious injuries like ACL tears requires a considerable amount of physical and mental energy from the athlete. In elite sport, periods of high stress are often followed by planned recovery in which the workload (intensity, volume and density) are decreased to promote positive physical adaptation and avoid maladaptation. Thus, an integral component of the return to sport performance plan shown here includes planning and periodization methodologies aimed at accounting for the stress of rehabilitation, the requirement to restore physical work capacity to pre-injury levels and avoid preventable setbacks.

### Neuromuscular Testing and Monitoring

#### Milestones and Progressions

There is no scientific consensus on a sport-specific return to sport protocol for alpine ski racers post-ACLR surgery. As such, the return to sport training plan and protocol presented in this case study were arrived at by the IPT based on the collective domain-specific expertise of the group, general recommendations in the scientific literature on return to sport protocols for ACL injured athletes, and an emerging body of knowledge generated by the IPT based on several years of consistent and routine monitoring of ACLR athletes. The objectives of the neuromuscular testing and monitoring approach were 5-fold: (1) use objectively determined, standardized and repeatable off-snow tests that reflect the demands of alpine ski racing; (2) employ a serial *athlete monitoring* approach vs. a single-time-point *clearance* approach; (3) use the results of the neuromuscular tests to drive the training and rehabilitation program design process; (4) spur better conversations amongst the IPT in an effort to manage the complexity and uncertainty of the return to sport process after ACLR; and (5) to assess and restore the basic functional properties of the neuromuscular system (e.g., maximal strength, RFD, the force-velocity relationship, the strength curve) alongside redeveloping movement competencies that were typically evaluated subjectively by the IPT members.

**Figure 4:**
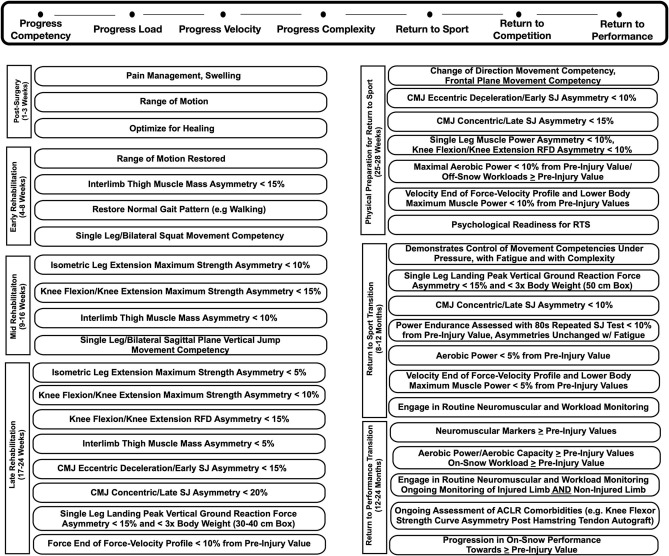
Target neuromuscular milestone and workload progressions post ACL reconstruction surgery. CMJ, Countermovement Jump; SJ, Squat Jump; RFD, Rate of Force Development; RTS, Return to Sport; Eccentrric Decel, Eccentric Deceleration.

Figure 4 depicts a framework for guiding the return to sport transition including consideration for tissue healing, movement competencies, functional milestones, and work capacity. The initial focus post-surgery included optimizing tissue healing, restoring range of motion, normalizing gait patterns, and increasing thigh muscle volume. Thigh muscle volume was assessed consistently throughout the return to sport training plan through anthropometric testing. This data is not presented. The athlete was sequentially cleared throughout the return to sport training plan, and progressively more challenging movement competencies were introduced, such as progressing from squatting and jumping in the sagittal plane to jumping in the frontal plane and then to high velocity change of direction movements.

The milestones achieved in the previous phase directed the IPTs strategy on an on-going basis. For example, maximal muscle strength (i.e., maximum muscle force) was trained and monitored at the end of each 4 week training period in the early and mid-phases of rehabilitation. Once maximal isometric strength asymmetries presented below 10%, higher velocity movement competencies in the sagittal and frontal plane were introduced. This highlights a significant difference between a time-based and milestone-based approach. Interlimb vertical jump asymmetry assessments were routinely performed on a dual force plate system and used to monitor functional recovery of the athlete. Importantly, numerous pre-injury neuromuscular testing sessions, including vertical jump asymmetry assessments, provided a baseline for comparison, which is important given that the non-surgical limb may also detrain consequent to an ACL injury. This further highlights the significance of, and rationale for, regularly conducting neuromuscular testing in athletes at risk for ACL injury.

Neuromuscular testing also included consideration for the effects of common comorbidities on muscle strength and power. For example, a semitendinosus tendon autograft is the most common ACLR surgical procedure employed with Canadian alpine skiers (Jordan et al., 2017c). And, this procedure is known to cause long-term hamstring strength deficits, especially at larger angles of knee flexion (>70° knee flexion) (Konrath et al., 2016). Thus, the strength curve of the hamstrings and quadriceps, and RFD ability, that is unique from the capacity to generate high muscle force, were routinely assessed throughout the return to sport and the return to performance phases (Jordan et al., 2015c). Prior to the return to sport transition, the athlete's workload capacity, aerobic power, and power endurance were assessed. Importantly, the IPT evaluated the stability of movement competencies and functional asymmetries when the athlete was under pressure (e.g., increased cognitive task demands) and fatigued. Finally, neuromuscular monitoring was conducted throughout the return to sport and return to performance phases, given the prevalence of ACL reinjury in the first 24 months post ACLR surgery.

#### Vertical Jump Force-Time Asymmetry Testing

**Figure 5:**
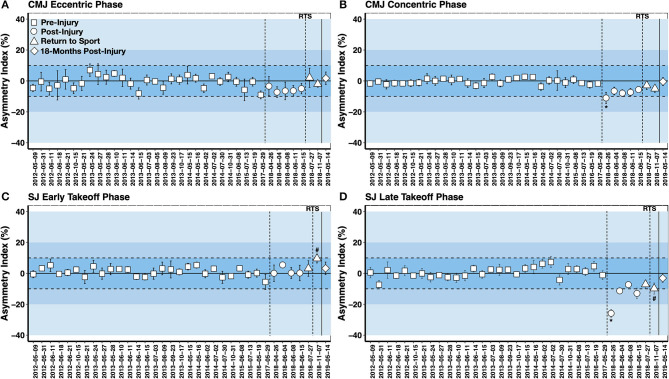
Squat jump (SJ) and countermovement jump (CMJ) force-time asymmetry assessments pre-injury, post-injury, at return to sport (RTS) and 18 months post-surgery (**A**: CMJ Eccentric Deceleration Phase; **B**: CMJ Concentric Phase; **C**: SJ Early Takeoff Phase; **D**: SJ Late Takeoff Phase). Data are shown as the five-jump mean asymmetry index ± SD (positive value = ACLR limb dominance; negative value = contralateral limb dominance). SJs demonstrating a small amplitude countermovement were discarded from the analysis. *First asymmetry test post-injury; # observation of shift in the interlimb asymmetry at teh return to sport phase.

Vertical jump force-time asymmetry testing on a dual force plate system was undertaken as a component of the routine athlete monitoring program both before and after injury (Figure 5). This assessment has been used previously with alpine ski racers (Jordan et al., 2015b, 2018), elite soccer players with lower body injury (Hart et al., 2019), and to evaluate neuromuscular function in ACLR athletes (Baumgart et al., 2017; Miles et al., 2019). The vertical jump force-time asymmetry assessment is a standardized and repeatable test that is easily employed in the daily training environment of elite athletes. It also permits an evaluation of eccentric vs. concentric movement abilities, and as eccentric movements occur frequently in alpine ski racing, the IPT could evaluate eccentric movement abilities throughout the return to sport training period in a standardized manner.

Figure 5 depicts the pre-injury and post-injury vertical jump force-time asymmetries. An elevated interlimb asymmetry index beyond this threshold was noted at the first testing point for the concentric phase of the CMJ and the late takeoff phase of the SJ (Asymmetry Index >25%). There was a steady recovery observed in the CMJ eccentric deceleration phase asymmetry index to baseline values at the return to sport transition. However, CMJ concentric phase and SJ late takeoff phase asymmetries remained elevated compared to baseline measurements until 18 months post-surgery. An unanticipated loading strategy was seen in the final SJ asymmetry test of the return to sport transition. Notably, increased asymmetry reflecting ACLR limb dominance was found in the early takeoff phase of the SJ, whereas increased asymmetry reflecting contralateral limb dominance was observed in the late takeoff phase of the SJ. A shift in the interlimb asymmetry throughout the range of motion of the vertical jump has been previously observed in ACLR alpine skiers (Jordan et al., 2015b). The reason for this is unknown, but it suggests that the force-time curve should be characterized in its entirety when evaluating vertical jump asymmetries. Further, contrary to evaluating limb symmetry in strength or RFD, the vertical jump can be considered a complex movement where no two jumps are the same and no jump is perfectly symmetrical. Thus, asymmetries may be best understood by characterizing the mean and variation over multiple movement cycles.

**Figure 6:**
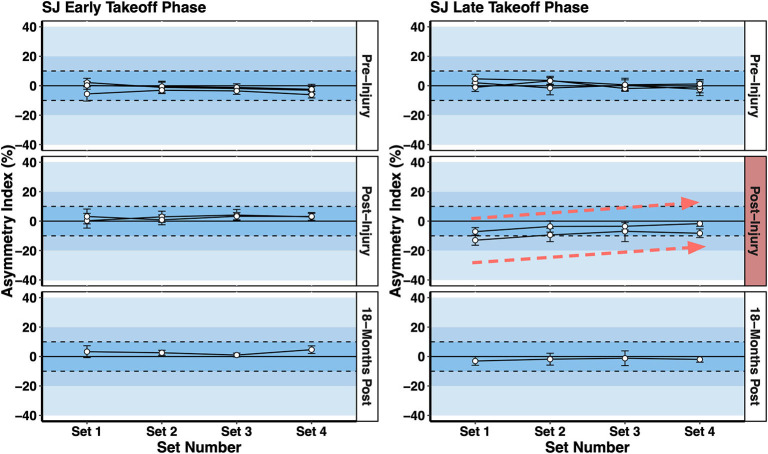
Squat jump (SJ) asymmetries in the early takeoff phase (initiation of jump to the peak vertical ground reaction force) and late takeoff phase (peak vertical ground reaction force to toe-off) over the 80 s repeated SJ test. A comparison is made between pre-injury tests and post-injury tests. A decline in late takeoff phase asymmetry (i.e. increased symmetry) with fatigue (i.e., Set 1 vs. Set 4) was observed post-injury reflecting a decline in force generated by the contralateral limb.

Vertical jump force-time asymmetries were also affected by fatigue in the 80 s repeated SJ test (Figure 6). This test was developed to evaluate an alpine ski racer's power-endurance and lower body RFD abilities over a time period that is similar to a typical training run or ski race (Jordan et al., 2017b). Not only can power-endurance and work capacity be assessed with this test but also the acute effects of fatigue on interlimb asymmetries.

A key observation shown in Figure 6 is that the interlimb asymmetry index in the *late takeoff phase* of the SJ declined with fatigue from > 10% in Set 1 (first 5 jumps at the start of the test) to <10% in Set 4 (last 5 jumps at the end of the test) for the two post-injury measurements, a pattern not observed in the pre-injury or 18 months post injury tests. This reflected the athlete becoming *more* symmetrical with fatigue due to a decrease in force generated by the non-surgical contralateral limb, presumably due to the acute effects of fatigue. There is no concrete scientific evidence to support a link between neuromuscular fatigue and risk for ACL injury. However, elite alpine ski racers and expert stakeholders rank fatigue as a contributing factor to injury (Spörri et al., 2012). Further, there is a higher prevalence of secondary ACL injury sustained by the contralateral limb in ACLR alpine ski racers compared to the ipsilateral limb (Pujol et al., 2007; Jordan et al., 2017c). There is no evidence to support a causal relationship between the observations presented in this case study and the potential for ACL reinjury. However, assessing work capacity, power-endurance, and ability to maintain movement competencies while fatigued, were important components of the return to sport training plan presented here.

#### Lower Body Mechanical Muscle Function, Maximal Muscle Strength, and Muscle Power

Lower body mechanical muscle function was assessed throughout the return to sport training plan including assessments of: (1) lower body maximal muscle power and power-endurance assessed in the CMJ and SJ; (2) lower body eccentric abilities including lower limb stiffness that measures the ability to reverse the downward acceleration of the BCM assessed in the CMJ; (3) knee extensor, knee flexor and leg press maximal strength and RFD ability evaluated during isometric MVCs; (4) CMJ takeoff velocity vs. load and eccentric deceleration impulse vs. load relationships; and (5) an evaluation of the knee flexor strength curve (i.e., torque-joint angle relationship) given the athlete underwent a semitendinosus tendon autograft, a procedure that is known to impact knee flexor torque at specific joint angles.

**Figure 7:**
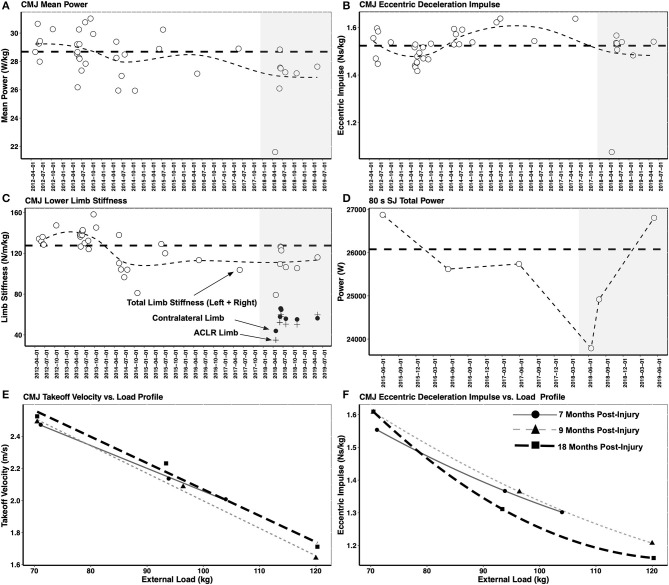
**(A–D)** Depict lower body mechanical muscle function assessed in the countermovement jump (CMJ) and squat jump (SJ). Shaded gray zone depicts the post-injury period. Thick horizontal dashed line shows the mean pre-injury value. Thin dashed line depicts trendline over the testing period. To highlight the importance of considering the strength of each limb independently alongside a measure of interlimb asymmetry, the ACLR limb stiffness (+ symbol) and non-injured limb stiffness (• symbol) are shown alongside the total limb stiffness (o symbol). While the interlimb asymmetry in limb stiffness was negligible at the 18 months post-injury time point, it can be seen that the total limb stiffness had still not fully recovered to the pre-injury mean value. **(E)** Shows the CMJ takeoff velocity vs. load profile and **(F)** shows the CMJ eccentric deceleration impulse vs. load profile in the post-injury testing period.

The aim of the testing battery was to assess a range of strength and movement abilities that are important for alpine ski racing performance using standardized and repeatable tests, including eccentric force-time abilities that have been shown to distinguish elite alpine ski racers from development-level skiers (Jordan et al., 2018). Further, the testing battery was also used to assess different muscle properties (force-velocity relationship, torque-joint angle relationship) with an aim to pinpoint deficits so that the rehabilitation and retraining could be tailored to the athlete's specific needs. Figure 7 depicts a selection of measures obtained from the CMJ and SJ analysis, including the loaded CMJ testing shown in the bottom two panels (Figures 7E,F). However, pre-injury measures are not shown for the loaded CMJ testing.

CMJ mean power and lower body limb stiffness were not restored to pre-injury levels at the 18 months post-injury timepoint. While the interlimb asymmetry in limb stiffness was negligible at the 18 months post-injury time point, it can be seen that the total limb stiffness had still not fully recovered to the pre-injury mean value. Only total power in the 80 s repeated SJ test (the sum of the mean power from all 20 jumps) and the CMJ net eccentric deceleration impulse were back to or above the pre-injury mean value at 18 months post-injury. However, the CMJ net eccentric deceleration impulse, representing the athlete's ability to reverse the downward acceleration of her BCM, may not reflect the demands of alpine ski racing given that elite alpine ski racers are exposed to near maximal eccentric loading exceeding body mass (Berg et al., 1995). Thus, the loaded CMJ protocol assessed the skier's ability to manage external loads greater than her body mass. At the 18 months post-injury testing timepoint (Figure 7F), while the unloaded CMJ eccentric deceleration impulse remained above the pre-injury mean value and the return to sport testing timepoint at 9 months post-injury, decreased force generating capacity for the high load condition was observed. This likely reflected a loss of maximal muscle strength, a finding that could be used to optimize the off-snow training period and the return to performance transition.

**Figure 8:**
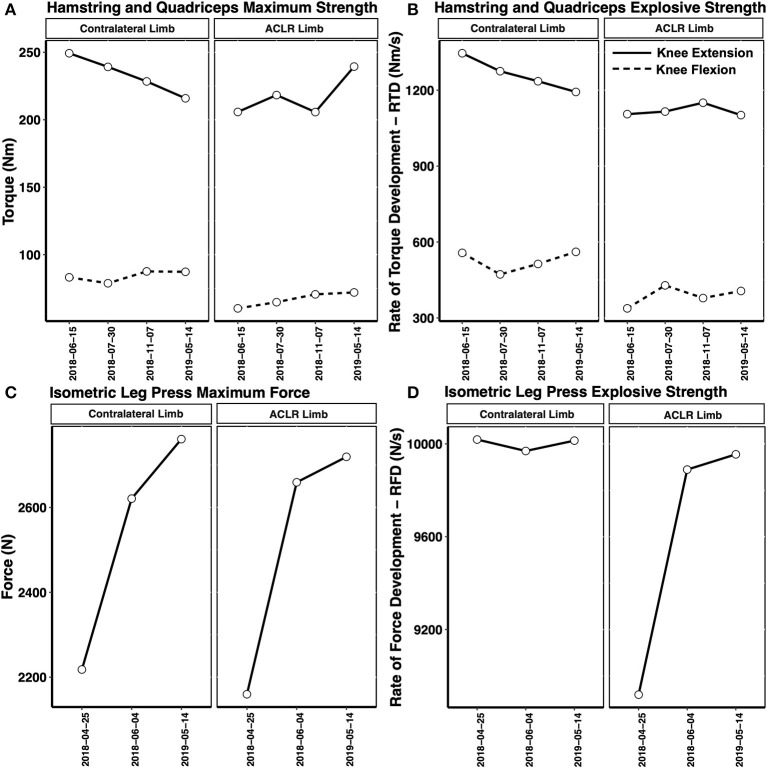
Recovery of the isometric knee extensor/knee flexor maximum torque **(A)**, rate of torque development—RTD **(B)**, leg press maximum force **(C)**, and leg press rate of force development—RFD **(D)** in the post-injury period. A decrease in the non-injured limb knee extensor maximal torque and RTD was observed through to the 18 months timepoint. Hamstring and quadriceps RTD remained depressed compared to maximal torque at 18 months post-injury. A small interlimb asymmetry in leg press maximum force and RFD was noted at 18 months post-injury.

Finally, MVCs of isometric leg press, isometric knee extension and isometric knee flexion were conducted throughout the return to sport training phase (Figure 8). Pre-injury values are not displayed as the tests were added after the injury was sustained. In addition to measuring the maximal leg press force and maximal knee extensor/flexor torque, an average slope analysis was conducted to assess the athlete's rapid force generating RFD ability. The physiological determinants of maximal muscle strength include muscle architecture (e.g., physiological cross sectional area—PCSA) and motor unit recruitment ability (Maffiuletti et al., 2016). While the physiological determinants of the late phase RFD (>100 ms from the onset of contraction) include factors influencing maximal muscle strength, neural factors such as the motor unit firing rate (rate coding) and doublet firing alongside the intrinsic muscle fiber properties are the primary determinants of early RFD (<100 ms from the onset of contraction) (Maffiuletti et al., 2016).

The recovery of leg press force, knee extensor torque and knee flexor torque are shown in Figure 8. Interestingly, the non-injured limb maximum knee extensor torque and RTD declined over the post-injury period, leading to a reduction in the interlimb asymmetry index. In fact, at the 18 months post-injury timepoint, the ACLR limb produced higher knee extensor torque compared to the non-injured limb (Asymmetry Index = 9%, reflecting ACLR limb dominance). Again, monitoring the neuromuscular function of both the ACLR limb and non-injured limb is critical. This observation further emphasizes the importance of evaluating the limb-specific torques, alongside the interlimb asymmetry index throughout the return to sport/return to performance transition, to ensure both limbs are sufficiently strong. Consistent with the scientific literature, a slower recovery in the knee extensor/knee flexor RFD ability was also noted throughout the post-injury period and return to sport transition. However, the leg press maximum force and RFD asymmetry indexes had recovered to below 3% by the 18 months post-injury testing session (Figures 8C,D).

## Discussion

The main objective of this paper was to present a return to sport training plan and return to sport clearance protocol, underpinned by neuromuscular testing and workload monitoring, for a female elite alpine ski racer who sustained an ACL injury. This paper also presented the functional role of the IPT, including practitioners in mental performance, sports medicine, orthopedic surgery, physiotherapy and physical performance sciences (S&C, sport science) in supporting the return to sport and return to performance transition after ACL injury.

The return to sport/return to performance transition after ACL injury is a multifactorial process characterized by complexity and many uncertainties (Buckthorpe, 2019; Burgi et al., 2019). It is of critical importance after ACLR surgery to ensure tissues have had sufficient time to heal, a process that often requires more time than is allotted by return to sport timelines (Nagelli and Hewett, 2016). To this end, time from surgery was considered in the return to sport training plan presented here. However, there are few pragmatic and concrete measures of healing, and time from surgery has little bearing on functional capacities after ACL injury (Barber-Westin and Noyes, 2011; Grindem et al., 2016; Burgi et al., 2019; Cristiani et al., 2019). Thus, objective neuromuscular testing is recommended after ACL injury to evaluate functional milestones alongside a carefully planned and progressive return to sport training plan (Myer et al., 2006; Grindem et al., 2016; Buckthorpe, 2019). This seems especially important for alpine ski racers with ACL injury given the high risk for traumatic injury (Flørenes et al., 2009; Bere et al., 2014), the high lower body strength and work capacity demands involved in ski racing (Berg et al., 1995), the frequency with which alpine skiers return to sport after ACL injury (Haida et al., 2016), and the prevalence of ACL reinjury (Pujol et al., 2007; Haida et al., 2016; Jordan et al., 2017c).

There are no evidence-based return to sport protocols or testing batteries for alpine ski racers with ACL injury, but developing this knowledge base is important (Jordan et al., 2017a). To this end, the return to sport testing protocol presented here included numerous tests that attempted to assess the movement competencies, strength abilities, and workload capacity of the athlete. While this protocol required specialized equipment and more time than traditional field testing, it was consistent with recommendations in the scientific literature of employing objectively determined tests that evaluate a range of neuromuscular abilities (Myer et al., 2006).

The neuromuscular testing battery included measures that have been shown to identify individuals at-risk for ACL reinjury, such as interlimb quadriceps strength symmetry (Grindem et al., 2016). Hamstring muscle strength was evaluated as well given the effects of the semitendinosus tendon autograft procedure on knee flexor torque (Konrath et al., 2016). Additionally, interlimb vertical jump asymmetries, permitting an evaluation of eccentric/concentric movement abilities, were evaluated using a dual force plate system. This approach has recently become more common for evaluating neuromuscular function in ACL injured athletes (Jordan et al., 2015b, 2018; Baumgart et al., 2017; Hart et al., 2019; Miles et al., 2019). The present protocol also conducted an assessment of power-endurance including the acute effects of fatigue on interlimb asymmetries. While there is no link between fatigue and the risk for ACL injury/reinjury, fatigue may affect the lower body force-generating capabilities of ACL injured athletes compared to non-injured controls (Jordan et al., 2017b). Consistent with this idea, the ACL injured athlete highlighted in this case study showed a reduction in SJ interlimb asymmetry with fatigue, reflecting less force generated by the non-injured limb in the final 20 s of the 80 s repeated squat jump test. This pattern was not observed at the 18 months post-injury timepoint and the total mechanical power produced in the 80 s SJ test had been restored above the pre-injury values by the final test.

In the present case study, the neuromuscular test battery appeared sensitive to the recovery process after ACL injury. Notably, the interlimb asymmetry index for the late takeoff phase of the SJ and concentric phase of the CMJ tracked most consistently with the time-course recovery following ACL injury (*c.f*. Figure 5). In support of this notion, the interlimb asymmetry indexes in these jump phases were previously shown to distinguish elite alpine ski racers with ACLR from skiers without ACLR (Jordan et al., 2015b). However, more importantly, the test battery allowed the IPT to pinpoint trainable deficits that could be addressed throughout the return to sport training plan. Additionally, an *athlete monitoring* strategy was employed vs. a single discrete time point *clearance* approach. The recovery after ACLR surgery is lengthy, often taking many years (Jordan et al., 2015a) and the prevalence of ACL reinjury is greatest in the first 2 years after ACLR surgery (Paterno et al., 2012, 2014). Thus, the return to sport transition, often occurring between 9 and 12 months after ACLR surgery, fails to account for the period with the highest risk of reinjury and the fact that ACLR athletes often wish to restore their pre-injury performance level (i.e., return to performance), a process that may require several years. The framework presented here attempted to account for the dynamic transitional nature of the recovery process after ACLR surgery, including consideration for the rehabilitation, return to sport and return to performance phases.

Despite the recommendation that objective testing be conducted in athletes with ACL injury to safeguard against reinjury, there appears to be limited evidence to support this notion (Burgi et al., 2019; Webster and Hewett, 2019). In fact, while passing commonly performed return to sport testing batteries was associated with a lower risk of ACL re-rupture, it was associated with a 3x higher risk for contralateral ACL injury (Burgi et al., 2019; Webster and Hewett, 2019). This highlights the importance of monitoring neuromuscular function in both the ACLR limb and non-injured limb alike throughout the return to sport/return to performance transition. In the present case study, the non-injured limb demonstrated a decline in quadriceps strength and RFD through to the 18-month post-injury time point.

Not only might RFD analysis provide additional insight into the recovery of an ACLR athlete, but it has also been shown that quadriceps and hamstring RFD remain persistently depressed in ACLR patients, recovering more slowly than maximum muscle strength. Active tissues, like skeletal muscle, including the quadriceps and hamstring muscles, are important for absorbing external energy to protect passive tissues like the ACL. Importantly, the hamstring muscle group acts as an ACL synergist counteracting anterior shear forces and tibial rotation that cause ACL strain (Barrata et al., 1988). Maximum muscle force is attained more than 500 ms after the onset of contraction, and as ACL injury events in alpine ski racing occur in much shorter time frames (i.e., <100 ms), hamstring RFD may be particularly important for providing dynamic knee joint stabilization to protect against ACL injury/reinjury (Jordan et al., 2015c). As functional testing like single leg hopping may be of little predictive value for identifying athletes at risk for ACL reinjury (Grindem et al., 2016), hamstring/quadriceps strength assessments, including assessing RFD ability, is important to evaluate in ACLR athletes throughout rehabilitation and the return to sport training phase. Despite observing a recovery in the interlimb vertical jump asymmetries, muscle strength and power deficits compared to the pre-injury mean value remained 18 months post-injury. This highlights the importance of baseline testing for athletes at-risk for ACL injury to provide benchmarks for comparison in the event of an injury. Based on the data presented here, a full recovery after ACL injury was not equated with a full return to sport and competition.

In addition to neuromuscular testing, return to sport protocols should also consider the psychological readiness of the athlete given its association with the risk for ACL reinjury (McPherson et al., 2019). Furthermore, the return to sport transition can be supported by a team approach that involves interdisciplinary expertise (Wang et al., 2019). Psychological readiness was not evaluated objectively in the present case study, which is a limitation especially given that alpine ski racing is an extreme sport with a high risk for traumatic injury (Flørenes et al., 2009; Bere et al., 2014), and that many factors related to skier psychology have been identified by expert stakeholders as contributors to injury risk (Spörri et al., 2012). Nevertheless, the IPT functioned as a *holacracy* or a flat leadership structure where the opinions of the domain-specific experts were considered equally, including those pertaining to the athlete's psychological readiness.

Finally, the present case study included an assessment of internal workload (sRPE) as a component of the return to sport training plan with an aim to ensure the athlete had sufficient work capacity and physical reserves to support the return to snow training camps. To the authors' best knowledge, there are currently no scientific investigations into the validity of the sRPE method for measuring the internal workload in alpine ski racers, and the relationship between work capacity and ACL injury/reinjury risk remains unknown.

## Limitations

There are limitations to the case study presented here. First, the generalizability of the present data to the elite alpine skiing population at large and to development-level alpine skiers is unknown. Scientific inquiry is also required to validate the proposed frameworks for safeguarding athlete health and safety following ACL injury. The physical demands of alpine ski racing, including the high forces and high velocities that are attained in an unpredictable environment (i.e., unknown and variable course conditions) are difficult to simulate in a controlled laboratory setting. Consequently, the neuromuscular tests that are proposed may be insufficient for evaluating the physical capacities of alpine skiers with ACL injury. To this end, wearable technologies such as inertial measurement units that can be worn during skiing may provide additional information to the IPT, including the ability to assess the relationship between off-snow neuromuscular testing indices and on-snow neuromuscular strategies that may be involved in ACL reinjury (Färber et al., 2018; Bessone et al., 2019). Finally, it is recognized that psychological readiness is an important determinant of return to sport readiness after ACL injury (McPherson et al., 2019). Psychological readiness was not assessed objectively in the present case study and this should be considered an important component of the return to sport transition after ACL injury given the extreme nature of alpine ski racing.

## Summary and Recommendations

Alpine ski racing is an extreme sport with a high risk for ACL injury and high proportion of skiers will return to sport post-ACLR surgery. Alpine skiers with ACL injury are at increased risk for ACL reinjury, and the physical and psychological readiness of alpine ski racers can be compromised after ACL injury. Return to sport after ACL injury is a complex and uncertain transitional period in an athlete's career and the interdisciplinary performance team (IPT) can support the recovery process through a team-approach to rehabilitation that is underpinned by athlete monitoring, neuromuscular testing, planning and science. However, more work remains to be done to establish a valid return to sport testing protocol and the training methods to support elite alpine ski racers with ACL injury. There are several potential recommendations and considerations that emerge from this case study alongside findings from the scientific literature that can assist practitioners in building a return to sport training plan for alpine skiers with ACL injury:

- Assemble team-oriented individuals including practitioners with expertise in sports medicine, physiotherapy, strength & conditioning, sport science/physiology, nutrition, biomechanics, and mental performance.
- Model the IPT off a *holacracy* whereby domain-specific-practitioners lead the discussion depending on the situation and the phase of the return to sport/return to performance transition. As return to sport after ACL injury is multifactorial, all members of the team, including the athlete, should feel empowered to communicate their concerns and opinions.
- Build a periodized return to sport training plan that follows accepted training principles, uses a functional milestone-based approach and details the phases through which the athlete will progress including the acute post-operative period, early/mid/late rehabilitation, return to sport transition and the return to performance transition. ACL reinjury typically occurs within the first 2 years post-injury so it is important that the return to sport training plan reflects this timeline.
- Alpine ski racers suffering ACL injury often sustain significant combined injury including multi-ligament injuries, meniscal tears and chondral injury. These factors delay typical return to sport timelines and should be factored into the return to sport training plan.
- The IPT should work closely with the coach to develop a progressive and appropriate return to snow plan that factors in the psychological readiness and physical readiness of the ACLR skier.
- Communicate the return to sport protocol in advance, prior to the occurrence of injuries, translating the concept of a milestone-based approach vs. time-based approach to coaches, athletes and members of the IPT.
- Start building an athlete-specific pre-injury profile, including baseline assessments of neuromuscular function, physical fitness, and workload capacity that can be used as benchmarks for comparison after ACL injury to guide the return to sport training plan.
- Workloads should be monitored and progressed throughout the return to sport transition so that an ACLR skier has sufficient physical reserves to endure the demands of return to snow training.
- A battery of standardized and reliable neuromuscular assessments that are objectively determined and specific to the demands of alpine ski racing should be employed to evaluate an alpine skier with ACL injury.
- Interlimb asymmetries are a useful indicator of recovery after ACL injury but it is important to ensure both limbs are sufficiently strong given that ACL injury impacts both the injured and non-injured limbs.
- Employ an *athlete monitoring* approach that tracks the athlete throughout the return to sport/return to performance transitions instead of a *discrete timepoint clearance* approach.

## Data Availability Statement

The datasets for this article are not publicly available because data pertains to Olympic athletes. Requests to access the datasets should be directed to MJ, mjordan@ucalgary.ca.

## Ethics Statement

The studies involving human participants were reviewed and approved by University of Calgary Research Ethics Board. The patients/participants provided their written informed consent to participate in this study. The animal study was reviewed and approved by University of Calgary Research Ethics Board.

## Author Contributions

MJ was responsible for preparing the manuscript. ML, NM, JB, MH and WH were responsible for data collection and data analysis. MH, SR and KM were responsible for medical milestones. All authors contributed to the concepts and framework presented in the manuscript.

### Conflict of Interest

The authors declare that the research was conducted in the absence of any commercial or financial relationships that could be construed as a potential conflict of interest.
